# Assessing Nursing Homes Quality Indicators’ between-Provider Variability and Reliability: A Cross-Sectional Study Using ICCs and Rankability

**DOI:** 10.3390/ijerph17249249

**Published:** 2020-12-10

**Authors:** Lauriane Favez, Franziska Zúñiga, Narayan Sharma, Catherine Blatter, Michael Simon

**Affiliations:** 1 Institute of Nursing Science, University of Basel, Bernoullistrasse 28, 4056 Basel, Switzerland; lauriane.favez@unibas.ch (L.F.); narayan.sharma@unibas.ch (N.S.); catherine.blatter@unibas.ch (C.B.); m.simon@unibas.ch (M.S.); 2 Nursing and Midwifery Research Unit, Inselspital Bern University Hospital, Freiburgstrasse, 3010 Bern, Switzerland

**Keywords:** nursing homes, long-term care, benchmarking, quality indicators, health care, quality of health care

## Abstract

Nursing home quality indicators are often used to publicly report the quality of nursing home care. In Switzerland, six national nursing home quality indicators covering four clinical domains (polypharmacy, pain, use of physical restraints and weight loss) were recently developed. To allow for meaningful comparisons, these indicators must reliably show differences in quality of care levels between nursing homes. This study’s objectives were to assess nursing home quality indicators’ between-provider variability and reliability using intraclass correlations and rankability. This approach has not yet been used in long-term care contexts but presents methodological advantages. This cross-sectional multicenter study uses data of 11,412 residents from a convenience sample of 152 Swiss nursing homes. After calculating intraclass correlation 1 (ICC1) and rankability, we describe between-provider variability for each quality indicator using empirical Bayes estimate-based caterpillar plots. To assess reliability, we used intraclass correlation 2 (ICC2). Overall, ICC1 values were high, ranging from 0.068 (95% confidence interval (CI) 0.047–0.086) for polypharmacy to 0.396 (95% CI 0.297–0.474) for physical restraints, with quality indicator caterpillar plots showing sufficient between-provider variability. However, testing for rankability produced mixed results, with low figures for two indicators (0.144 for polypharmacy; 0.471 for self-reported pain) and moderate to high figures for the four others (from 0.692 for observed pain to 0.976 for physical restraints). High ICC2 figures, ranging from 0.896 (95% CI 0.852–0.917) (self-reported pain) to 0.990 (95% CI 0.985–0.993) (physical restraints), indicated good reliability for all six quality indicators. Intraclass correlations and rankability can be used to assess nursing home quality indicators’ between-provider variability and reliability. The six selected quality indicators reliably distinguish care differences between nursing homes and can be recommended for use, although the variability of two—polypharmacy and self-reported pain—is substantially chance-driven, limiting their utility.

## 1. Introduction

Quality indicators are used worldwide to monitor, assess and report the quality of care provided in healthcare settings by measuring specific health care structures (e.g., staffing), processes (e.g., patient referrals) or outcomes (e.g., nosocomial infections) that reflect quality of care [1,2]. Healthcare providers can use them for continuous quality monitoring or for benchmarking, i.e., to compare healthcare providers, to measure quality against accepted standards or to measure developments over time. Benchmarking these indicators allows evaluation and comparison of healthcare providers’ quality of care levels. However, concerns have been expressed regarding the value of quality indicators and publicly reported benchmarking [3,4,5,6]. Therefore, to ensure that quality indicators provide useful information, they need to be evaluated by criteria including but not limited to validity, feasibility and relevance. Particularly in the context of publicly reported benchmarking, quality indicators have to reliably assess differences in quality of care between healthcare providers. Quality indicators should thus be able to show (1) between-provider variability and (2) reliability. Between-provider variability refers to the quality indicator’s ability to indicate differences in quality of care beyond chance, i.e., to identify higher-performing and lower-performing healthcare providers [7]. The quality indicator’s reliability is its capacity to accurately and consistently measure the particular quality it indicates [8,9]. If both characteristics apply to the quality indicators used, they can be used for benchmarking, which has the potential to support the maintenance and improvement of quality of care [10].

In several countries (e.g., the United States, Australia, Canada), nursing home quality indicators have been measured and reported publicly for some time [11]. Quality indicators cover a wide variety of themes, most commonly physical restraints, falls, pressure ulcers and weight loss. They tend to be assessed either with routinely used instruments (e.g., Resident Assessment Instrument-Minimum Data Set (RAI-MDS) in the United States and Canada) or through specific data collections (e.g., the National Aged Care Mandatory Quality Indicator Program in Australia). Depending on the country, the results may be reported to the nursing home administration, regionally and/or nationally [12,13,14,15]. In Switzerland, despite legislation providing legal bases for the measurement and public reporting of quality indicators in nursing homes since 1994, their measurement at the national level started only in 2019, with the first quality indicator results still not published as of October 2020 [16]. Their selection and development included a review and expert consultations, considering a variety of criteria (e.g., relevance, feasibility, reproducibility) [17,18]. In 2016, based on a broad stakeholder consultation, the first set of six quality indicators indicating percentages of specific health processes or outcomes among nursing home residents were selected: the percentage of residents with polypharmacy (one quality indicator), experiencing pain (two quality indicators), being subjected to physical restraints (two quality indicators) and with weight loss (one quality indicator). The details of the selection process are reported in Appendix A. After the selection was made, we conducted an analysis to evaluate whether the six quality indicators’ between-provider variability and reliability were adequate for national and publicly reported benchmarking.

Methods for assessing between-provider variability include intraclass correlation 1 (ICC1) and rankability. To assess each indicator’s capacity to differentiate between facilities, we used the ICC1, which reflects “the proportion of variance that is accounted for by the group level” [19]. While this method has been used in other settings to evaluate quality indicators’ between-provider variability (e.g., hospital quality indicators), it has not yet been used in long-term care contexts [20]. A second metric to assess between-provider variability is rankability, i.e., “the part of heterogeneity between … clinics [or nursing homes] that is due to true differences” [21]. Finally, it is also important to assess the group mean via intraclass correlation 2 (ICC2), which describes the reliability of each quality indicator [22]. Therefore, this study aims to use ICC1 and rankability to report on the six selected nursing home quality indicators’ between-provider variability and ICC2 to report on their reliability.

## 2. Materials and Methods

### 2.1. Design and Sample

This multicenter pilot study used routine resident data from a convenience sample of 152 nursing homes located across Switzerland’s three major language regions (German, French, Italian). In 2017, 1561 Swiss nursing homes provided both medical care and social services to more than 157,716 older adults. Around 80% of these people are long-term residents, with an average length of stay of 2.5 years [23]. Inclusion criteria at the nursing home level were for each facility to be licensed as a nursing home and to have agreed to work with a specific version of the assessment instrument including the items needed for the six national quality indicator measurements (Appendix B for further information). At the resident level, all long-term residents residing in the nursing home at the date of the data export were included.

### 2.2. Variables and Measurements

Each resident’s birth year (YYYY format), admission date to the nursing home (YYYYMMDD format), sex (male/female) and care level (scale of 1–12) were used. Care level—calculated based on an assessment performed by the nursing home staff—was allocated a number from 1 to 12, with each higher number representing an additional 20 min of care time per day. We calculated length of stay in days from admission to data export day, and residents’ age as the difference in years between birth year and year of the assessment. We also collected variables specifying each patient’s depressive (depression rating scale; DRS) and cognitive status (cognitive performance scale; CPS). The DRS is calculated on a scale from 0 to 14: scores of 3 or above indicate evidence of minor to major depression [24]. The CPS is calculated on a scale from 0 (“intact”) to 6 (“very severe impairment”) [25]. The size of the nursing home (number of beds) was also included.

We used the following variables to calculate the six quality indicators, all of which were collected for the recall period “in the last 7 days”: number of active ingredients taken; frequency and intensity of self-reported and observed pain; frequency of trunk fixation use or seating that prevents the residents from rising; frequency of bedrail use; and percentage of weight loss during the last 30 or 180 days. We also used a variable specifying whether the resident’s latest assessment was that at admission (yes/no) and whether the nursing home staff evaluated that he or she had a life expectancy of under 6 months (yes/no/information not collected). In case of use of a physical restraint, we also used a variable specifying the context in which the measure was applied (use of the measure on the request or in agreement with a resident capable of judgment/use of the measure on a resident incapable of judgment/context not yet clarified).

The six selected quality indicators were defined as follows: *polypharmacy* is the percentage of residents who took 9 or more active ingredients over the last 7 days. The cut-off value of 9 is in line with other international measures of polypharmacy in nursing homes [26]. *Pain* is measured by 2 quality indicators: *self-reported pain* is the percentage of residents with daily moderate or higher pain intensity or those with nondaily very strong pain intensity in the last 7 days. *Observed pain* is the percentage of residents who showed daily moderate or higher pain intensity or those who showed nondaily very strong pain intensity in the last 7 days. *Physical restraint* is also measured through 2 quality indicators. The first measures the percentage of residents with daily fixation of the trunk or with seating that prevented them from rising in the last 7 days; the second measures the percentage of residents with daily use of bedrails or other devices on all open sides of their bed so that they could not leave the bed independently in the last 7 days. The sixth quality indicator is *weight loss*, measuring the percentage of residents with weight loss of ≥5% in the last 30 days or of ≥10% in the last 180 days.

Quality indicators are described in a numerator/denominator format. Their results are expressed as a rate for each nursing home (e.g., percentage of residents with observed pain in a specific nursing home). The numerator includes all residents for whom the outcome of interest (e.g., pain) is indicated; the denominator includes all residents except those who fit predetermined exclusion criteria. For the self-reported pain quality indicator, for example, residents were excluded if they did not give a valid answer to questions related to pain frequency or intensity. For both physical restraint quality indicators, residents capable of judgement who either requested or agreed to the measure were excluded. For the weight loss quality indicator, we applied two exclusion criteria: resident’s life expectancy estimated by the staff to be under 6 months or current assessment of the resident is the admission assessment. Definitions, numerators, denominators, items measured and exclusion criteria for all quality indicators are displayed in Table 1.

### 2.3. Data Collection

Resident data were collected by the nursing homes via updated versions of routinely used resident assessments instruments between July 2016 and August 2017. All quality indicator information was obtained through routine data collection processes already in place, including observations (e.g., physical restraints) or conversations with residents (e.g., self-reported pain). At the time of the study in Switzerland, three assessment instruments were in use: (1) Nursing Home Resident Assessment Instrument (RAI-NH), (2) the Planification Informatisée des Soins Infirmiers Requis (PLASIR/PLEX) (computerized planning of required nursing care) and (3) the BewohnerInnen-Einstufungs-und Abrechnungssystem (BESA) (residents classification and billing system) [27]. Further information on these instruments is available in Appendix B. Each assessment instrument provider had to recruit a minimum of 50 nursing homes to ensure the sample would have an equal number of nursing homes working with each instrument. The goal was to have a total study sample consisting of minimum 10% of all Swiss nursing homes. To ensure that all residents present in each nursing home at the time of the data export were assessed at least once, data were collected for a minimum of 6 months in each nursing home.

### 2.4. Statistical Analysis

We examined the data on each quality indicator for completeness, plausibility and missing values. Missing data were dealt with by listwise deletion; for each quality indicator, the number of valid residents depended on the exclusion criteria. We also computed resident characteristics and prevalence rates for the six studied quality indicators. Risk adjustment for the indicators was assessed in a preparatory study using hierarchical multiple regression models, with the Akaike information criteria and odds ratios assessed for each quality indicator. All indicators were risk-adjusted for the resident’s cognitive performance and care dependency with additional adjustment for polymedication with age and for both pain indicators with depression and gender. Risk adjustment variables are provided in Table 2. Statistical analyses were conducted by N.S., confirmed by C.B. and supervised by M.S., who has a track record of statistical analyses in healthcare quality measurement.

#### 2.4.1. Between-Provider Variability: ICC1 and Rankability

To assess each quality indicator’s capacity to distinguish between providers, we computed ICC1, caterpillar plots and rankability. The ICC1 shows the proportion of variation in the quality indicator that is attributed to the group level [28]. In this context, ICC1 values typically range from 0.0 to 0.3, where values over 0.05 indicate relevant between-provider variability [19,29,30]. The ICC1 is the ratio of variance among providers (*VG*) over the total variance, i.e., the group variance (*VG*) and the within-group or residual variance (*VR*). As we analyze binary outcomes, VR is the latent scale variance of the logit model *π*^2^/*3*, leading to the following equation: *ICC1* = *VG* / (*VG* + *π*^2^/*3*) [29,31].

#### 2.4.2. Reliability: ICC2

Variances for the ICC1 were calculated using a conditional generalized linear mixed model with 95% confidence intervals to assess the uncertainty of the estimate [32]. Additionally, to check the distribution of nursing home estimates and explore between-provider variability visually, we computed caterpillar plots based on empirical Bayes estimates with 95% confidence intervals [33,34]. Each quality indicator’s caterpillar plot shows that indicator’s estimate for each nursing home (e.g., weight loss), as well as whether it deviates positively or negatively from the grand mean across all nursing homes. We calculated ICC1 figures with the rptR package in R (Version 3.6.6., R Core Team, 2020) [31,34]. We also explored quality indicators’ rankability, i.e., the part of variability between nursing homes measured by quality indicators that results from true differences in quality of care [35]. High rankability for a particular indicator allows performance ranking for that indicator, e.g., polymedication [20]. Rankability (ρ) is defined as: ρ = *VG*/(*VG* + *median*(*s*^2^)), with *median*(*s*^2^) indicating the variance of the individual facility effect estimates from a fixed effect regression model. Rankability (range: 0–100%) refers to observed differences that might result from quality of care disparities and is classed as low (<50%), moderate (50–75%) or high (>75%) [28,35]. Finally, we assessed the six quality indicators’ group mean reliability via ICC2. ICC2 is the ratio of group variance to total variance/*k*, where *k* is the number of nursing homes, i.e., *ICC2* = *VG*/((*VG*+ *π*^2^/*3*) × (*1*/*k*)) [19].

ICC1 and ICC2 are generally interdependent: the higher a quality indicator’s ICC1, the higher its ICC2. The ICC2 typically ranges from 0.6 to 1.0, with values closer to 1 indicating higher measurement reliability.

### 2.5. Data Management and Ethical Considerations

At the end of the data collection period, each instrument developer anonymized all resident-level data and transferred all records to the Swiss Federal Office of Public Health, which pseudonymized them at the nursing home level. The study data were then transferred to the research group, which carried out the analysis. Data protection and confidentiality were ensured during every phase of the study. The Ethics Committee of Northwest and Central Switzerland declared that according to Swiss legislation, the study did not require ethical clearance (EKNZ Req-2017-00052).

## 3. Results

### 3.1. Sample and Quality Indicators Description

A total of 152 nursing homes participated in the study (56 for RAI-NH, 46 for PLAISIR/PLEX, 50 for BESA; mean size: 102.3 beds (standard deviation (SD): 51.2)). These housed 11,412 residents (mean age: 86.1 years (SD: 8.36); 72.8% female). The median length of stay was 859 days (interquartile range (IQR): 375–1646 days), with a median care level of 6.0 (IQR: 4–9). The mean prevalence of each quality indicator and missing item data per quality indicator are displayed in Table 2.

### 3.2. Between-Provider Variability: ICC1 and Rankability

**Figure 1:**
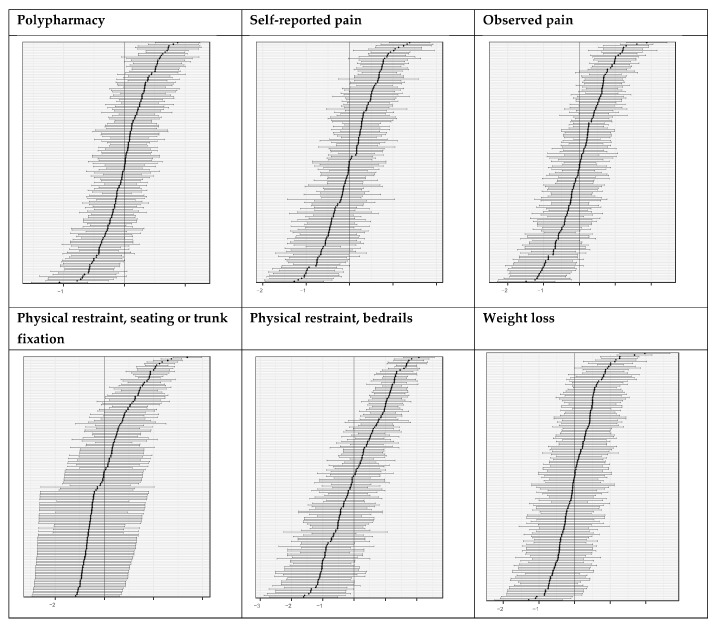
Caterpillar plots based on empirical Bayes estimates of the six quality indicators. In each plot, the horizontal lines represent the nursing homes: the dot in the middle of each line represents the percentage of nursing home residents to whom the quality indicator applies; the whiskers represent the 95% confidence interval (CI). The vertical line represents the (standardized, i.e., centered to 0) sample mean of the specified quality indicator. If the whiskers (i.e., the CI) do not touch the vertical line (i.e., the mean), the result of the nursing home in the quality indicator specified (e.g., the percentage of residents with weight loss in this nursing home) differs significantly from the sample mean (e.g., the mean percentage of residents with weigh loss across all nursing homes). Lower-performing nursing homes with quality indicator values above the mean, e.g., more residents with weight loss, are on the top-right side of the plot. Those with quality indicator values below the mean, e.g., fewer residents with weight loss, are on the negative (lower left) side of the plot. If the CI touches the sample mean, the nursing home’s result does not differ significantly from the mean.

The between-provider variability of this study’s six selected quality indicators was relatively high: all ICC1 values were above 0.05, ranging from 0.068 (polypharmacy) to 0.396 (physical restraint, trunk fixation or seating that prevents the resident from rising). Our caterpillar plots illustrate that each quality indicator can discriminate sufficiently between the higher- and lower-performing nursing homes (Figure 1). On the one hand, we were able to identify better-performing nursing homes, i.e., those housing submean proportions of residents with the indicator result; e.g., 17 nursing homes had significantly fewer residents with polypharmacy. Only in the case of the *physical restraint,* *trunk fixation or seating that prevents the resident from rising* quality indicator was it not possible to differentiate higher-performing nursing homes, as many facilities had no such cases, resulting in a low mean. On the other hand, we were able to identify lower-performing nursing homes, i.e., those having significantly higher proportions of residents with the quality indicator result than the mean; e.g., 13 had a higher percentage of residents with weight loss. Rankability values ranged from low, at 0.144 (polypharmacy) and 0.471 (self-reported pain); to moderate, at 0.692 (observed pain) and 0.720 (weight loss); to high, at 0.865 (physical restraint, bedrails) and 0.976 (physical restraint, trunk fixation or seating that prevents the resident from rising). ICC1 and rankability results are provided in Table 3. Unadjusted results are provided in Appendix C.

### 3.3. Reliability: ICC2

The reliability results of the six quality indicators were high according to usual standards: ICC2 ranged from 0.896 (self-reported pain) to 0.990 (physical restraint, trunk fixation or seating that prevents the resident from rising). ICC2 results are found in Table 2. Unadjusted results are provided in Appendix C.

## 4. Discussion

For each of six selected quality indicators, this study uses ICC1, rankability and ICC2 to evaluate two important properties: between-provider variability and reliability. Our results show that four of the six quality indicators (observed pain, physical restraint, trunk fixation or seating that prevents the resident from rising and bedrails, malnutrition) have high ICC1, moderate to high rankability and high ICC2 values. This indicates respectively that between-nursing home variability was high and that these four quality indicators were generally reliable. Two indicators—polypharmacy and self-reported pain—also showed high reliability and variability beyond chance, however to a lesser extent, which makes them less ideal for comparing nursing homes. These two quality indicators represent similar challenges for nursing homes. Even with focused efforts, polypharmacy is difficult to tackle for facilities and reducing the polypharmacy rate might be complex because of structural circumstances (e.g., physician system, legal regulations). Similarly, lowering the percentage of residents with pain is complex (i.e., difficulties in and possibilities for treating chronic painful conditions) for nursing homes. Therefore, from a measurement viewpoint, while four quality indicators can be recommended without hesitation for publicly reported benchmarking, two do not fully achieve this status.

Internationally, reports of nursing home quality indicators’ between-provider variability are rare. To our knowledge, the study by Rantz et al. (2004), who reported a between-provider variability evaluation for 23 nursing home quality indicators used in the United States, is the only published study to do so. That study grouped nursing homes according to resident outcomes, tested the groups for significant differences and performed pairwise comparisons [36]. Of the 23 quality indicators tested, the authors concluded that only ten could distinguish the group of nursing homes with good resident outcomes from that whose corresponding outcomes—including for polymedication and weight loss—were poorer. In contrast, ICC1 and rankability provide measures to assess each indicator’s ability to differentiate between facilities. ICC1 does so while addressing clustering and multiple testing, neither of which featured in the study by Rantz et al. However, Rantz et al. were by no means exceptional in this respect: our literature review could not identify a single study using ICC1, rankability or caterpillar plots based on empirical Bayes estimates to evaluate between-provider variability in the long-term care sector, although all have been used in other fields.

Among publicly reported nursing home performance figures, a small number of countries (e.g., the Netherlands) have reported the reliability of isolated quality indicators, while Germany and the United States have published studies or reports assessing the reliability of entire nursing home quality indicator sets (respectively, of 10 and more than 100 quality indicators) [37,38,39,40]. These studies used single item-level and/or weighted kappas and percent agreement between “gold standard” nurses and nursing home nurses to assess the selected quality indicators’ interrater and intrarater reliability. Reliability results varied widely between quality indicators. The use of Cohen’s kappa to assess nursing home quality indicators’ reliability only provides information on the reliability of individual measures. Using ICC2 allows us to acquire information on the reliability of quality indicators at the group level, which we argue is more interesting, as the facility mean is targeted rather than the reliability of the measure at the individual level [29]. While this level of reference makes the ICC2 ideal for nursing homes benchmarking, we could identify no other studies using it as a reliability measure.

Despite having widely different cut-off values, our results show that ICC1 and rankability correlated strongly: high ICC1 values were reflected by high rankability values; e.g., our highest ICC1 value, 0.396, was linked to our highest rankability value, 0.976 (for physical restraint, trunk fixation or seating that prevents the resident from rising). The same applies for low figures: our lowest ICC1, 0.068 corresponded with the lowest rankability, 0.144 (for polypharmacy). This relationship has been explored with a similar correlation for the ranking of binary hospital quality indicators [20]. Although an ICC1 of 0.05 has been regarded as the lower threshold for quality indicators, considering the rankability found in our study, this threshold might be higher, at roughly 0.15. Indeed, if both rankability and ICC1 results are higher than the threshold, there is evidence of differences in quality of care between nursing homes, and these quality indicators can therefore be recommended.

Several issues surround the use of quality indicators that have not been adequately evaluated or simply do not meet acceptable standards. Quality indicators that cannot distinguish quality of care differences are not usable to publicly benchmark healthcare providers: they can lead to the publication of erroneous information, inappropriate comparisons or misguided quality improvement efforts, i.e., resulting from nursing home administrators’ or policy-makers’ use of them to set quality improvement targets. Further, inaccurate benchmarking results can lead to unjustified rewards or sanctions both by governments and by other stakeholders, particularly residents’ families [3,41]. Conversely, regular, accurate reporting on meaningful quality indicators contributes to accountability and transparency in the healthcare system [10]. Therefore, ensuring nursing home quality indicators’ can identify between-provider variability and reliability for benchmarking is a highly important step in their evaluation.

Although quality indicators can be extremely useful to identify quality improvement targets at the nursing home and policy levels, they cannot be used without considering the context. For example, as each quality indicator shows only one very limited aspect of a healthcare provider’s care, no single quality indicator can be used to characterize providers’ overall quality of care. Instead, sets of reliable quality indicators can show nursing home administrators their facilities’ rankings compared to other providers, thereby allowing them to identify, prioritize and allocate resources to quality improvement targets. However, while quality indicators are excellent tools for comparing quantifiable outcomes, they do not identify poor results’ underlying problems, indicate whether results are clinically meaningful or guide nursing homes regarding their improvement (i.e., regarding which specific factors require action or at which levels) [42,43]. Perhaps most importantly, while well-developed and well-evaluated quality indicators can provide valuable information to nursing homes, nursing home administrators often lack the skills, knowledge, leadership or professional and organizational capacities to put that information to good use [43]. Therefore, nursing homes need to work towards developing feasible strategies to identify and act on genuine quality improvement efforts based on quality indicator results.

Despite this pilot study’s large sample size, which includes around 10% of all Swiss nursing homes (1561 nursing homes in Switzerland in 2017), the included nursing homes’ mean bed count (102 beds) was somewhat higher than the Swiss average (62 beds) [23]. Even based on this rather homogeneous sample in comparison to the full sample of Swiss nursing homes, all six selected quality indicators showed between-provider variability, indicating they could be used in Swiss nursing homes. The indicator of self-reported pain had 13.4% missing values due to respondents not wanting or not being able to answer, which precludes a proportion of nursing home residents from being represented by this indicator. Therefore, it is important to evaluate both self-reported and observed pain, since the latter includes all residents. We report ICC1 and ICC2 in this study. While the value of the ICC1 is unchallenged in assessing between-provider variability, the ICC2 has generally been less frequently used and more recent literature has identified difficulties of the ICC2 when ICC1 values are very low [44]. This is not the case in our study; however, the generally high ICC2 values might indicate less sensitivity. The rankability scores seem to provide a more nuanced picture.

## 5. Conclusions

Based on the six selected nursing home quality indicators’ ICC1, rankability and ICC2 values, we determined that all six quality indicators can reliably distinguish differences in quality of care between nursing homes, although two operate at a lower level. Even though all are suitable as quality indicators for benchmarking and public reporting, for two of them, the observed variability is substantially driven by chance, limiting their utility. Still, they can serve nursing homes to assess their quality in this area and initiate quality improvement projects where needed. This pilot study showed that both ICCs and rankability are meaningful methods both to evaluate nursing home quality indicators’ between-provider variability and reliability and to validate them. Assessing quality indicators’ measurement properties is an essential step towards building sets of quality indicators that are useful in nursing home practice, policy and research. Public reporting of quality indicators increases transparency of the quality of care provided in nursing homes and provides an assessment of the national system. In practice, such indicators allow nursing homes to compare themselves with other facilities. For lower-performing nursing homes, this can be a starting point in identifying domains where quality improvement might be needed. Identifying higher-performing nursing homes helps to identify best care practices in these domains and enable learning from them. Regular evaluation of nursing home quality indicators, including between-provider variability and reliability, should be carried out and reported in all applicable contexts.

## Figures and Tables

**Table 1:** Description of the six Swiss quality indicators.

Theme	Definition	Numerator	Denominator	Items Measured	Exclusion Criteria
**Polypharmacy**	Percentage of residents who took 9 or more active ingredients in the last 7 days	All residents who had taken 9 or more active ingredients in the last 7 days	All long-term care residents	Number of active ingredients in the last 7 days	No exclusion criteria
**Self-reported pain**	Percentage of residents with daily moderate or higher pain intensity or residents with nondaily very strong pain intensity in the last 7 days	All residents who reported the following pain in the last 7 days: - Daily moderate, strong or very strong, unbearable pain OR - Nondaily very strong, unbearable pain	All long-term care residents, excluding those who did not give a valid answer regarding frequency or intensity of self-reported pain	Frequency and intensity of self-reported pain in the last 7 days	No valid answer to questions on frequency OR intensity of self-reported pain
**Observed pain**	Percentage of residents who showed daily moderate or higher pain intensity or residents who showed nondaily very strong pain intensity in the last 7 days	All residents where the following pain was observed in the last 7 days: - Daily moderate, strong or very strong, unbearable pain OR - Nondaily very strong, unbearable pain	All long-term care residents	Frequency and intensity of observed pain in the last 7 days	No exclusion criteria
**Physical restraint, trunk fixation or seating that prevents the resident from rising**	Percentage of residents with daily fixation of the trunk or with seating that prevented the resident from rising in the last 7 days	All residents who had daily in the last 7 days: - Trunk fixation OR - Seating that prevents the resident from rising	All long-term residents, excluding those who wanted or agreed to the use of this measure	Frequency of use in the last 7 days and context of the measure	Residents capable of judgment who either requested or agreed to the measure
**Physical restraint, bedrails**	Percentage of residents with daily use of bedrails or other devices on all open sides of the bed that did not allow the resident to leave the bed independently in the last 7 days	Residents with daily application of bedrails or other devices on all open sides of the bed, which does not allow the resident to leave the bed independently	All long-term residents, excluding those who requested or agreed to the use of this measure	Frequency of use in the last 7 days and context of the measure	Residents capable of judgment who either requested or agreed to this measure
**Weight loss**	Percentage of residents with weight loss of 5% or more in the last 30 days or of 10% or more in the last 180 days	Residents with a weight loss of 5% or more in the last 30 days or 10% or more in the last 180 days	All residents, excluding those with a life expectancy estimated by the staff as lower than 6 months or residents who were last assessed at admission to the nursing home	Weight loss of 5% or more in the last 30 days or of 10% or more in last 180 days	Residents with: - Life expectancy under 6 months - Last assessment at admission

**Table 2:** Risk adjustment variables, prevalence rates and missing values for the six Swiss quality indicators.

Theme	Risk Adjustment Variables	Prevalence Rate, Mean %, SD ^1^	Missing, % (n)
**Polypharmacy**	- CPS ^2^ - care level - age	43.0 (12.9)	0.0 (0)
**Self-reported pain**	- CPS - care level - depression - gender	19.7 (11.8)	13.4 (1525)
**Observed pain**	- CPS - care level - depression - gender	14.9 (10.4)	0.7 (81)
**Physical restraint**, **trunk fixation or seating that prevents the resident from rising**	- CPS - care level	3.4 (5.2)	0.0 (0)
**Physical restraint**, **bedrails**	- CPS - care level	13.0 (11.3)	1.6 (132)
**Weight loss**	- CPS - care level	7.9 (6.8)	0.1 (2)

Abbreviations: ^1^ SD: standard deviation, ^2^ CPS: cognitive performance scale.

**Table 3:** Risk-adjusted results of intraclass correlation 1 (ICC1), intraclass correlation 2 (ICC2) and rankability of the six quality indicators.

Theme	ICC1 ^1^ (95% CI ^2^)	ICC2 ^3^ (95% CI)	Rankability (ρ)
Polypharmacy	0.068 (0.047–0.086)	0.917 (0.889–0.935)	0.144
Self-reported pain	0.134 (0.104–0.166)	0.896 (0.852–0.917)	0.471
Observed pain	0.223 (0.131–0.325)	0.941 (0.879–0.965)	0.692
Physical restraint, trunk fixation or seating that prevents the resident from rising	0.396 (0.297–0.474)	0.990 (0.985–0.993)	0.976
Physical restraint, bedrails	0.371 (0.297–0.425)	0.989 (0.984–0.991)	0.865
Weight loss	0.137 (0.085–0.180)	0.899 (0.856–0.922)	0.720

Abbreviations: ^1^ ICC1: intraclass correlation 1, ^2^ CI: confidence interval, ^3^ ICC2: intraclass correlation 2.

## Appendix A

### Selection Process of the Four Themes of the Swiss Quality Indicators

The selection of the four themes covered by the six Swiss quality indicators was based on a multistep method-guided process started in 2008 [17]. This process was managed by a Steering Committee under the leadership of the Swiss association of nursing homes (CURAVIVA), with the participation of the Swiss Federal Office of Public Health, the Swiss Conference of Cantonal Health Directors and the Swiss Federal Statistical Office. The first step consisted of a literature review on the themes measured by quality indicators in the nursing home sector at the international, national and cantonal levels [45,46,47,48]. Based on this review, the Steering Committee selected five themes: physical restraint, weight loss, behavioral and psychological symptoms of dementia, medication (polypharmacy and antipsychotics) and pain.

The following phase consisted of the development of definitions, the development of evidence-based measurements and the operationalization of the measurements and answer options for the five selected themes. According to the RAND/UCLA methodology, development was supported by a multiexpert consultation for each theme chosen [49]. In 2014, the results of this development and of the quality indicator definitions at the time were sent to the appropriate offices of the Swiss federal government, cantons, nursing home associations, professional societies and associations, insurance companies, assessment instruments and other experts for consultation [50]. The Steering Committee then selected the 6 quality indicators with the highest acceptance rate (based on the national consultation) and prepared them for national measurement. The six selected quality indicators cover 4 themes: physical restraint (2 quality indicators), weight loss, polypharmacy and pain (2 quality indicators). Criticism of the nonselected themes included doubts about their validity as quality indicators (e.g., antipsychotic use) or the fear that some themes, if used, could send misleading signals (e.g., behavioral and psychological symptoms of dementia). The topics chosen show similarities to the quality indicator set recommended at roughly the same time in Germany [51]. Furthermore, based on a literature review, the Steering Committee also recommended variables to be tested for exclusion criteria and risk adjustment. Switzerland’s approach is to start with a limited set of well-evaluated quality indicators, then to strengthen and complete this set over time with additional quality indicators.

## Appendix B

**Table A1:** Overview of the assessment instruments used in the pilot study.

Assessment Instrument ^1^	Nursing Home Resident Assessment Instrument	Planification Informatisée des Soins Infirmiers Requis ^2^	BewohnerInnen-Einstufungs-und Abrechnungssystem ^3^
**Abbreviation**	RAI-NH	PLAISIR/PLEX	BESA
**Distributor in Switzerland**	Q-Sys	Eros	BESAcare
**Language availability**	German, French, Italian	French	German, French, Italian
**QI variables integration**	Updated version of the instrument	Additional module	Updated version of the instrument
**Data collection by**	Healthcare staff	Healthcare staff or external evaluators (choice of each NH)	Healthcare staff
**Start of the data collection (month)**	July 2016	July 2016	July 2016
**Data export (month)**	August 2017	February 2017	August 2017

^1^ At the time of the study, only three assessment instruments were in use by Swiss nursing homes. Each nursing home has the right to choose a preferred assessment instrument. These are used to carry out routine resident data collection to help evaluate aspects of residents’ needs and care (e.g., amount of care needed, cognitive functions, mobility). One such assessment must be carried out at each resident’s admission, then at least once every 6 months. The data gathered serve as a basis for care planning and health insurance claims [27]. ^2^ Computerized planning of required nursing care. ^3^ Residents classification and billing system.

## Appendix C

**Table A2:** Unadjusted results of intraclass correlation 1 (ICC1), intraclass correlation 2 (ICC2) and rankability of the six quality indicators.

Theme	ICC1 (95% CI)	ICC2 (95% CI)	Rankability (ρ)
Polypharmacy	0.055 (0.037–0.068)	0.898 (0.865–0.918)	0.120
Self-reported pain	0.119 (0.087–0.149)	0.953 (0.931–0.962)	0.437
Observed pain	0.147 (0.113–0.177)	0.963 (0.949–0.971)	0.575
Physical restraint, trunk fixation or seating that prevents the resident from rising	0.343 (0.235–0.405)	0.988 (0.980–0.991)	0.970
Physical restraint, bedrails	0.245 (0.197–0.286)	0.980 (0.973–0.983)	0.783
Weight loss	0.135 (0.095–0.165)	0.959 (0.941–0.969)	0.715

Abbreviations: ICC1: intraclass correlation 1, CI: confidence interval, ICC2: intraclass correlation 2.
